# Inhibition of histone H3K79 methylation selectively inhibits proliferation, self-renewal and metastatic potential of breast cancer

**DOI:** 10.18632/oncotarget.2496

**Published:** 2014-11-10

**Authors:** Li Zhang, Lisheng Deng, Fengju Chen, Yuan Yao, Bulan Wu, Liping Wei, Qianxing Mo, Yongcheng Song

**Affiliations:** ^1^ Department of Pharmacology, Baylor College of Medicine, Houston, TX 77030, USA; ^2^ Dan L. Duncan Cancer Center, Baylor College of Medicine, Houston, TX 77030, USA; ^3^ Department of Medicine, Section of Hematology/Oncology, Baylor College of Medicine, Houston, TX 77030, USA

**Keywords:** DOT1L, histone methylation, inhibitor, breast cancer, cancer stem cell

## Abstract

Histone lysine methylation regulates gene expression and cancer initiation. Bioinformatics analysis suggested that DOT1L, a histone H3-lysine79 (H3K79) methyltransferase, plays a potentially important role in breast cancer. DOT1L inhibition selectively inhibited proliferation, self-renewal, metastatic potential of breast cancer cells and induced cell differentiation. In addition, inhibitors of S-adenosylhomocysteine hydrolase (SAHH), such as neplanocin and 3-deazaneplanocin, also inhibited both H3K79 methylation and proliferation of breast cancer cells in vitro and in vivo. The activity of SAHH inhibitors was previously attributed to inhibition of H3K27 methyltransferase EZH2. However, inhibition of EZH2 by a specific inhibitor did not contribute to cell death. SAHH inhibitors had only weak activity against H3K27 methylation and their activity is therefore mainly due to DOT1L/H3K79 methylation inhibition. Overall, we showed that DOT1L is a potential drug target for breast cancer.

## INTRODUCTION

Post-translational modifications such as methylation of histone lysine side chains play important roles in gene regulation and dysfunction of histone methylation often leads to diseases such as cancer [1–4]. Histone H3-lysine79 (H3K79) methylation has been recently found to be a marker for active gene transcription from yeast to mammals [5–7]. DOT1L (disruptor of telomeric silencing 1 like), a human homolog of DOT1 in yeast, is the only histone methyltransferase that produces methylated H3K79 (H3K79me_x_, x = 1, 2 or 3) (7, 8). Given the crucial roles of H3K79 methylation in transcriptional elongation, DOT1L's function is tightly regulated: DOT1L has been found to be assembled as a member of several large transcription protein complexes containing transcription factors AF4, AF9, AF10 and ENL [9–13], which might control the location and extent of H3K79 methylation across the human genome.

**Figure 1:**
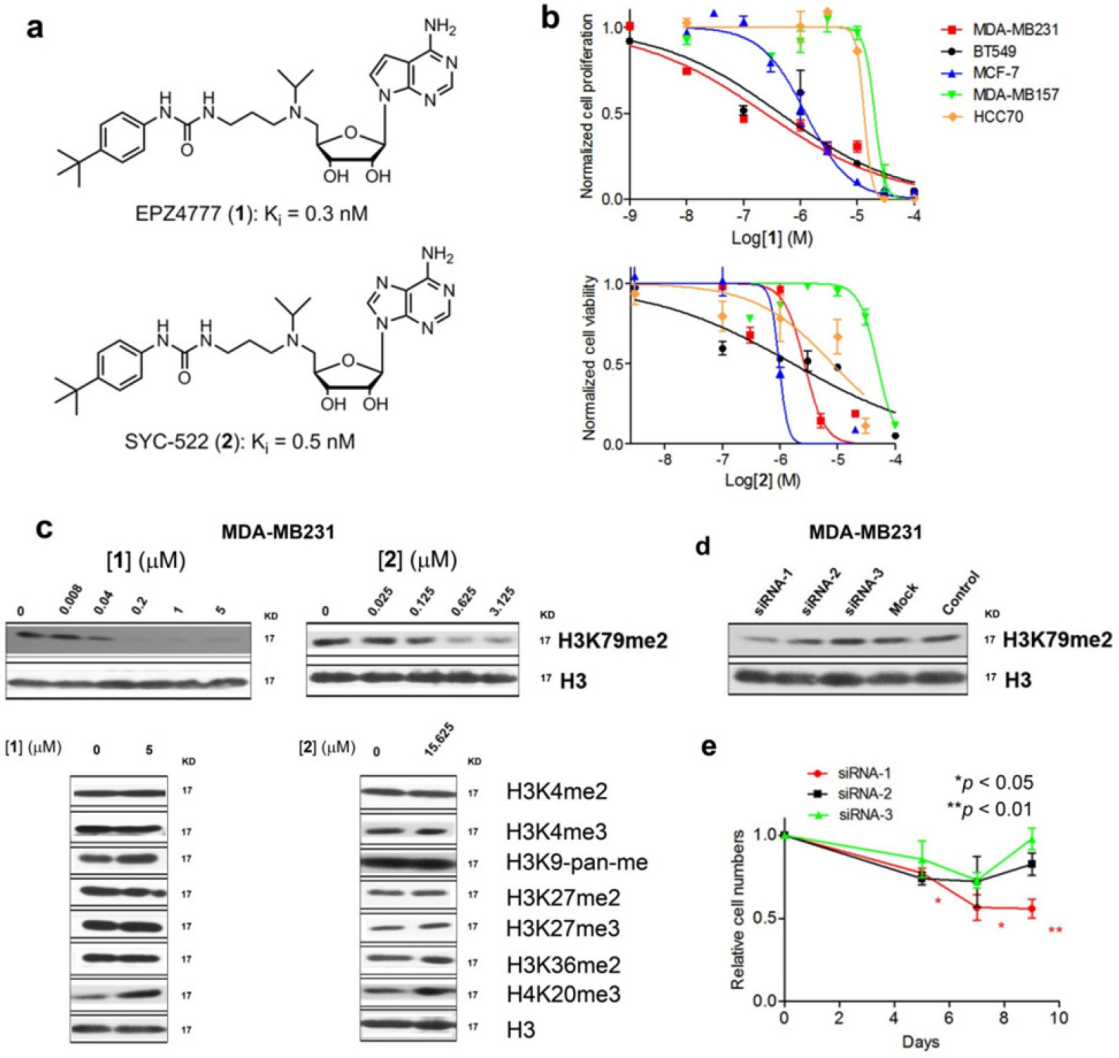
DOT1L inhibitors inhibited H3K79 methylation and proliferation of certain breast cancer cells (**a**) Structures of compounds **1** and **2**; (**b**) Dose response curves of compounds **1** (above) and **2** against five breast cancer cell lines, from which EC_50_ values were calculated; (**c**) Western blot results showing that compounds **1** and **2** only inhibited H3K79 methylation in MDA-MB231; (**d, e**) siRNA-1 significantly reduced H3K79 methylation in MDA-MB231. It also significantly inhibited cell proliferation. siRNA-2 and -3 had considerably weaker inhibition of H3K79 methylation and did not significantly affect the growth of MDA-MB231. (Statistical analysis by Student's *t* test: *p < 0.05, **p < 0.01)

DOT1L is a drug target for mixed lineage leukemia (MLL) gene rearranged leukemia [14–16]. The onco-MLL loses its C-terminal segment and is fused with a partner protein, e.g., AF4, AF9, AF10 or ENL, which can recruit DOT1L, causing abnormal H3K79 methylation. This leads to overexpression of a number of MLL target genes (e.g., HoxA9 and Meis1) and eventually the initiation of leukemia. EPZ004777 (**1**, developed by Epizyme, Inc.) [16] and SYC-522 (**2**, by us) [17] shown in Figure 1a were recently reported to potently inhibit DOT1L (*K*_i_: 0.3 and 0.5 nM, respectively), with >1,000-fold selectivity over other HMTs. These DOT1L inhibitors also exhibit selective activity against MLL-rearranged leukemia [16–18].

Despite the crucial roles of H3K79 methylation in gene regulation, few publications have linked DOT1L to other diseases to date [19]. A recent paper describes small interfering RNA (siRNA) mediated DOT1L knockdown inhibited proliferation of lung cancer cell lines, such as A549 [20]. However, we found that DOT1L inhibitors **1** and **2** had negligible activity against A549 cells (described below). Here, we report that DOT1L is a novel drug target for breast cancer. DOT1L/H3K79 methylation inhibition selectively inhibited proliferation, induced differentiation, and reduced cell migration and invasiveness of breast cancer cells with a relatively high DOT1L.

## RESULTS

### DOT1L was suggested to link to breast cancer

Exploring a breast cancer genomic database (https://genome-cancer.ucsc.edu/) covering >1,000 patient samples including 100 normal breast tissues [21], we found higher expression of DOT1L is significantly correlated with breast cancer (*p* < 0.001) as compared to normal breast tissues (Supplementary Fig. 1). Moreover, higher DOT1L levels in ~50% breast cancers correlate with overexpression of ~20 pro-proliferation genes in the left-panel of the PAM50 gene set (*p* < 0.001) (Supplementary Fig. 2). Overexpression of these genes links to high proliferation and poor prognosis of breast cancer [22]. These results suggested that the function(s) of DOT1L could be important to breast cancer.

Analysis also showed DOT1L expression levels are significantly correlated with estrogen receptor negative (ER^−^) breast cancers (Supplementary Fig. 3A), although a smaller proportion of ER^+^ breast cancers still express relatively high levels of DOT1L. In addition, DOT1L is not significantly correlated with expression of human epithelial growth factor receptor 2 (HER2) (Supplementary Fig. 3B), a biomarker for another clinically important subtype of breast cancer.

### DOT1L inhibition selectively inhibited proliferation of DOT1L^+^ breast cancer cells

We tested the activity of DOT1L specific inhibitors **1** and **2** against a panel of five breast cancer cell lines. Also included in the study were lung cancer A549 and non-MLL leukemia NB4 cells. Both compounds exhibited no or negligible activity (EC_50_ ≥ 15 μM) against all of these cells during a 3-day treatment (Table 1), showing they do not have non-specific cytotoxicity. For a 15-day treatment, these two inhibitors had strong activity against proliferation of MDA-MB231, BT549 (both showing ER^−^) and MCF-7 (ER^+^) breast cancer cells expressing relatively high levels of DOT1L (Table 1) [21], with EC_50_ values of 0.19 – 1.4 μM (Fig. 1b). The slow anti-proliferation activity for the DOT1L inhibitors was also observed in previous studies against MLL leukemia [16, 17]. Compounds **1** and **2** exhibited only weak activity (EC_50_: 12 – >50 μM) against breast cancer cells MDA-MB157 and HCC70 with low DOT1L expression levels as well as non-breast cancer cells A549 and NB4.

**Table 1:** Anti-proliferative activity (μM) of DOT1L inhibitors.^a^

	DOT1L level^b^	1 (EPZ004777)		2 (SYC-522)	
		3-day	15-day	3-day	15-day
MDA-MB231	0.39	45	0.19	>50	1.3
BT549	0.32	29	0.71	>50	1.4
MCF-7	0.12	15	0.95	41	0.91
MDA-MB157	−0.36	>50	27	>50	44
HCC70	−0.44	20	12	>50	18
A549	N.D.^c^	19	20	>50	>50
NB4	N.D.^c^	21	22	>50	>50

a EC_50_ values are the mean values from at least three independent experiments.

b DOT1L expression data are from the UCSC database (Zhu, et al., 2009).

c N.D., not determined.

To see if DOT1L inhibition is responsible for the anti-proliferation activity, compounds **1** and **2** were found to only inhibit H3K79 methylation in MDA-MB231 cells with IC_50_ values of ~50 and 100 nM, respectively (Fig. 1c). Next, three siRNAs targeting DOT1L were used to treat MDA-MB231 cells. siRNA-1 significantly reduced H3K79 methylation and also slowed down cell proliferation (Fig. 1d/e). Two other siRNAs as well as a negative control siRNA that did not significantly decreased H3K79 methylation levels had insignificant activity. To exclude possible off-target effects, one additional siRNA was found to be able to reduce H3K79 methylation as well as cell proliferation (Supplementary Fig. 4a/b) during the revision of the manuscript. Similar activities were observed for ER^+^ MCF-7 cells (Supplementary Fig. 4c-e). Treatment of MCF-7 cells with compound **1** resulted in considerably reduced H3K79 methylation in a dose-dependent manner. DOT1L knockdown by three siRNAs also led to decreased H3K79 methylation as well as inhibited cell proliferation. Overall, these observations showed that H3K79 methylation is important for DOT1L^+^ breast cancer (regardless of ER status) and DOT1L inhibition selectively inhibits proliferation of these cells.

### DOT1L inhibition reduced self-renewal and promoted differentiation

**Figure 2:**
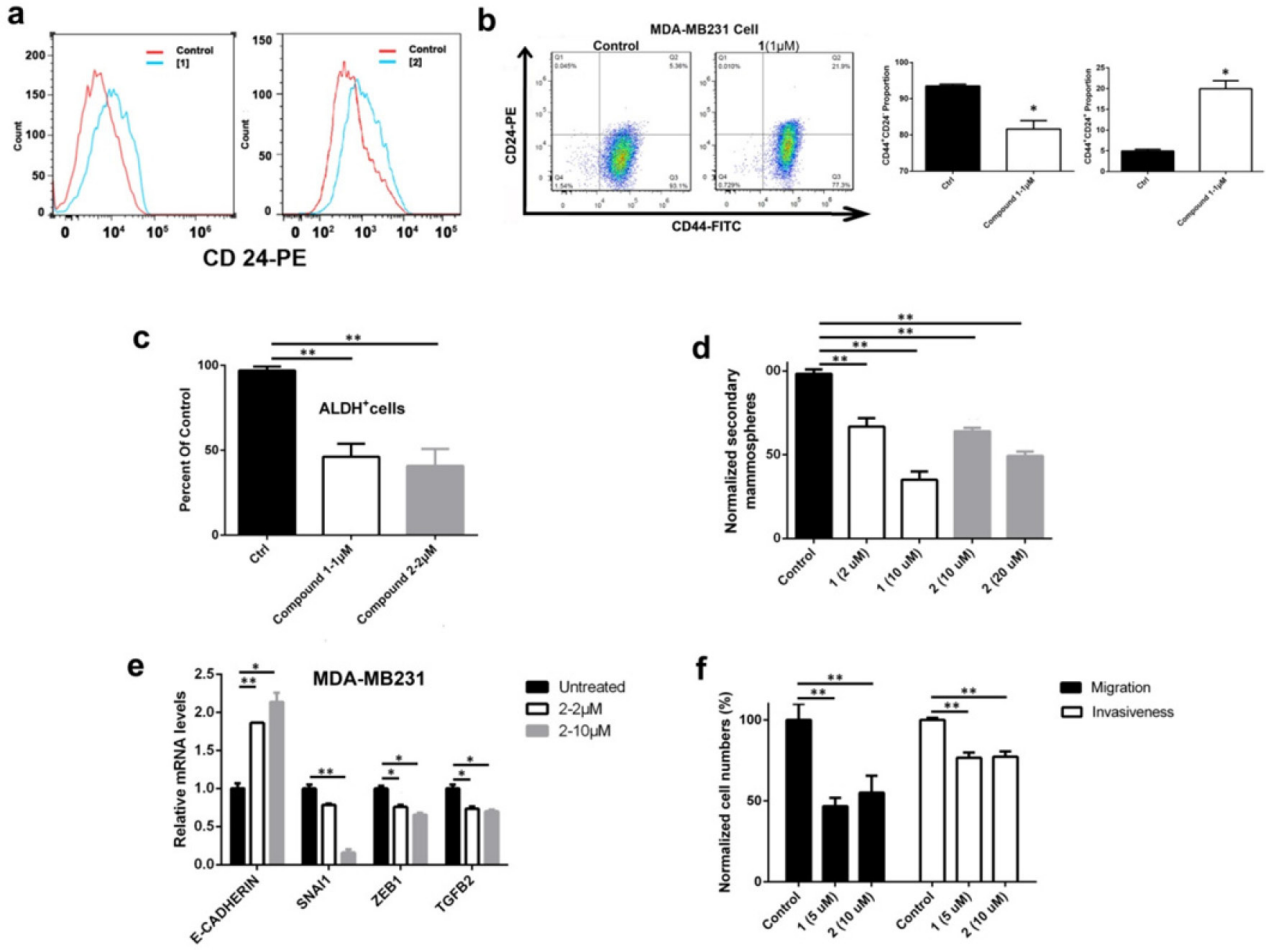
DOT1L inhibition induced cell differentiation, inhibited stem-like cells and cell migration/invasion, and corrected dysregulated gene expression of MDA-MB231 cells (**a**) Treatment with **1** and **2** caused an increased cell population expressing high levels of CD24, a protein characteristic to breast luminal cells, showing DOT1L inhibition induced cell differentiation; (**b**-**d**) Treatment with DOT1L inhibitors significantly (**b**) decreased the CD44^+^/CD24^−^ and increased the CD44^+^/CD24^+^ cell population; (**c**) reduced ALDH^+^ cell population, and (**d**) decreased the number of secondary mammospheres, suggesting that DOT1L inhibition impaired breast CSC-like cells; (**e**) qPCR experiments showed that DOT1L inhibition caused upregulation of E-cadherin as well as downregulation of Snail1, Zeb1 and TGF-β, indicating inhibited EMT; (**f**) Treatment with **1** and **2** reduced abilities of cell migration and invasion in Transwell microplates with 8 μm pores coated with Matrigel. (Statistical analysis by Student's *t* test: *p < 0.05, **p < 0.01)

Treatment of MDA-MB231 cells with compound **2** at 2 μM caused a negligible amount of apoptosis. At a higher concentration of 10 μM, it induced a modest apoptotic rate of ~8%, which is consistent with the result of cell cycle assay showing a moderately increased pre-G phase cell population (Supplementary Fig. 5a/b). These results suggested that the DOT1L inhibitors inhibit cell proliferation with a different mechanism from cytotoxic chemotherapeutics. We next tested whether these compounds have activity against cancer stem cells (CSCs), which are a small fraction of cancer cells that have certain stem cell traits (e.g., self-renewal and ability to differentiate) and can initiate new tumors when transplanted to a new host. CSCs have been known to be resistant to cytotoxic drugs that target rapidly dividing cancer cells and therefore believed to be responsible for drug resistance and tumor recurrence. Due to heterogeneity of breast cancers, there have been debates on what cell markers characterize breast CSCs [23, 24]. Nevertheless, many studies showed that breast CSCs are enriched in the CD44^+^/CD24^−/low^ subpopulation [25] as well as that with a high expression of aldehyde dehydrogenase (ALDH^+^) [26], which plays an important role in maintaining stem/progenitor cells. Upon treatment of MDA-MB231 cells with **1** and **2**, fluorescence-activated cell sorting (FACS) analysis showed that considerably more cells expressed higher levels of CD24 (Fig. 2a), a cell surface protein characteristic to breast luminal epithelial cells [27], showing these compounds can induce differentiation of CSC-like cells in mesenchymal MDA-MB231 cells. The CD44^+^/CD24^−^ cell population was significantly decreased (Fig. 2b and Supplementary Fig. 5c). ALDEFLUOR experiments (Fig. 2c and Supplementary Fig. 5d/e) showed that treatment with compounds **1** and **2** considerably reduced the ALDH^+^ cell population. These results suggested that these compounds can reduce the number of CSC-like cells by inducing cell differentiation. Moreover, compounds **1** and **2** can inhibit the ability of MDA-MB231 to form secondary mammospheres, a method to characterize the number of CSC-like cells in breast cancer cells [28], by 30 - >60% in a dose dependent manner (Fig. 2d). All these results supported that DOT1L inhibition induced differentiation, impaired self-renewal ability, and reduced the number of CSC-like cells.

### DOT1L inhibition impaired EMT and metastatic potential

In addition to rendering drug resistance, CSC is closely linked to epithelial mesenchymal transition (EMT) and metastasis [29, 30]. EMT, characterized by high expression of TGF-β, Snail, Zeb genes as well as low expression of cell-cell adhesion genes such as E-cadherin, is important for cancer cells to be migratory, invasive and generate metastasis. Using quantitative PCR (qPCR), incubation of MDA-MB231 cells with compound **2** can significantly reduce gene expression of TGF-β, Snail and Zeb, as well as increase that of E-cadherin in a dose-dependent manner (Fig. 2e), showing the ability of the DOT1L inhibitor to inhibit EMT. Impaired EMT by DOT1L inhibition was also demonstrated by in vitro cell migration and invasion assays. DOT1L inhibitors **1** and **2** can inhibit cell migration as well as Matrigel invasion of MDA-MB231 by ~50% and 25% (Fig. 2f), respectively, indicating the potential of these compounds to inhibit tumor metastasis.

### Microarray studies of DOT1L inhibition

**Figure 3:**
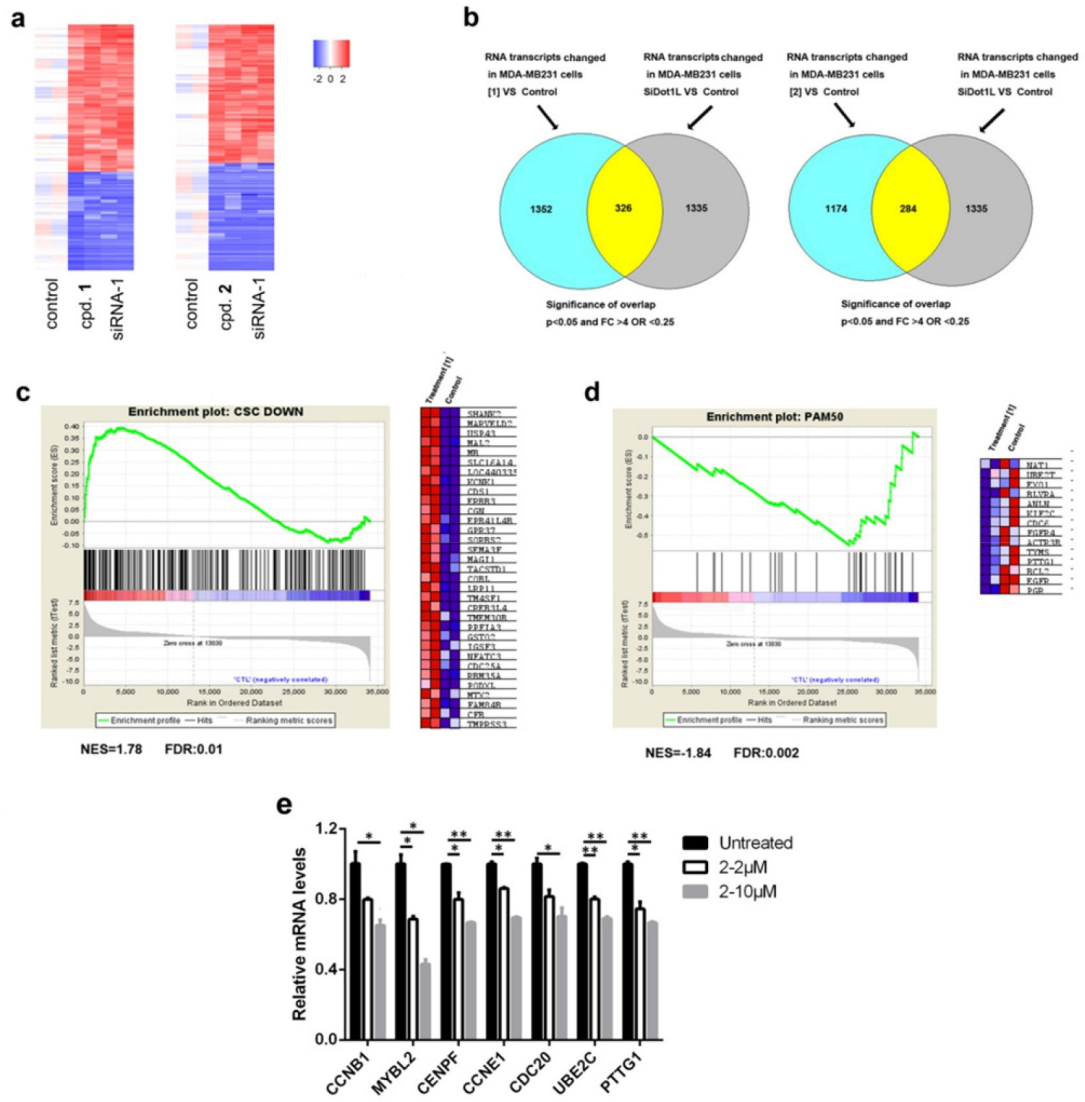
Microarray and qPCR results of DOT1L inhibition in MDA-MB231 cells (**a**) Heat maps of the overlapping upregulated and downregulated genes between samples treated with compounds **1** (5 μM) or **2** (10 μM) and those with siRNA-1 (duplicate samples, *p* < 0.05, fold change > 4); (**b**) Venn diagram showing numbers of significantly changed genes between samples treated with **1** or **2** and those with siRNA-1 (*p* = 1E-14 or 1E-16, respectively, one-sided Fisher's exact test); (**c**, **d**) Upon treatment with compound **1** (5 μM), GSEA plots showed (**c**) upregulation of the genes whose expression is low in breast CSCs and (**d**) downregulation of the pro-proliferation genes in the PAM50 gene set. The right panels in (**c**) and (**d**) are heat maps showing expression levels of selected genes in the leading edges of the GSEA plots; (**e**) qPCR experiments showed that DOT1L inhibition caused downregulation of pro-proliferation genes in breast cancer. (Statistical analysis by Student's *t* test: *p < 0.05, **p < 0.01)

Given that H3K79 methylation is a master regulator for gene transcription, a whole-genome profiling was performed to determine how DOT1L inhibition changes gene expression, in order to find the mechanism by which these compounds reduce proliferation of breast cancer cells. RNA was isolated from MDA-MB231 cells treated with siRNA-1 and compounds **1** and **2**, amplified, and hybridized to Illumina HT-12 microarrays. The data were log2-transformed and normalized to have the same median value for all arrays. Moderate *t*-test was used to examine whether genes were differentially expressed between the controls and treatments, with the filter thresholds being *p-*values < 0.05 and fold changes >4. As compared to the control samples, treatments with compounds **1**, **2** and siRNA-1 were found to cause highly similar changes in gene expression pattern (Fig. 3a/b and Supplementary Fig. 6a/b) (*p* = 10^−14^ and 10^−16^), indicating that the observed activities of the two compounds were mediated by DOT1L inhibition. Gene set enrichment analysis (GSEA) showed that treatment with the DOT1L inhibitors caused significant up-regulation of the gene set whose expression is low in breast CSCs (31) (Fig. 3c and Supplementary Fig. 6c). These results were consistent with our experimental observations and supported that DOT1L inhibition impairs CSCs.

In addition, as shown in Fig. 3d and Supplementary Fig. 6d, treatment with the DOT1L inhibitors led to significant down-regulation of the pro-proliferation genes in the PAM50 gene set, which were found to be overexpressed in breast cancer and link to aggressiveness and poor prognosis of breast cancer [22]. Among these, down-regulation of seven genes (*CCNB1, MYBL2, CENPF, CCNE1, CDC20, UBE2C* and *PTTG1*) was confirmed by qPCR (Fig. 3e). These results suggested that down-regulation of these pro-proliferation genes of breast cancer contributes to the anti-proliferation activity of the DOT1L inhibitors.

### SAHH inhibitors selectively inhibited DOT1L/H3K79 methylation

**Figure 4:**
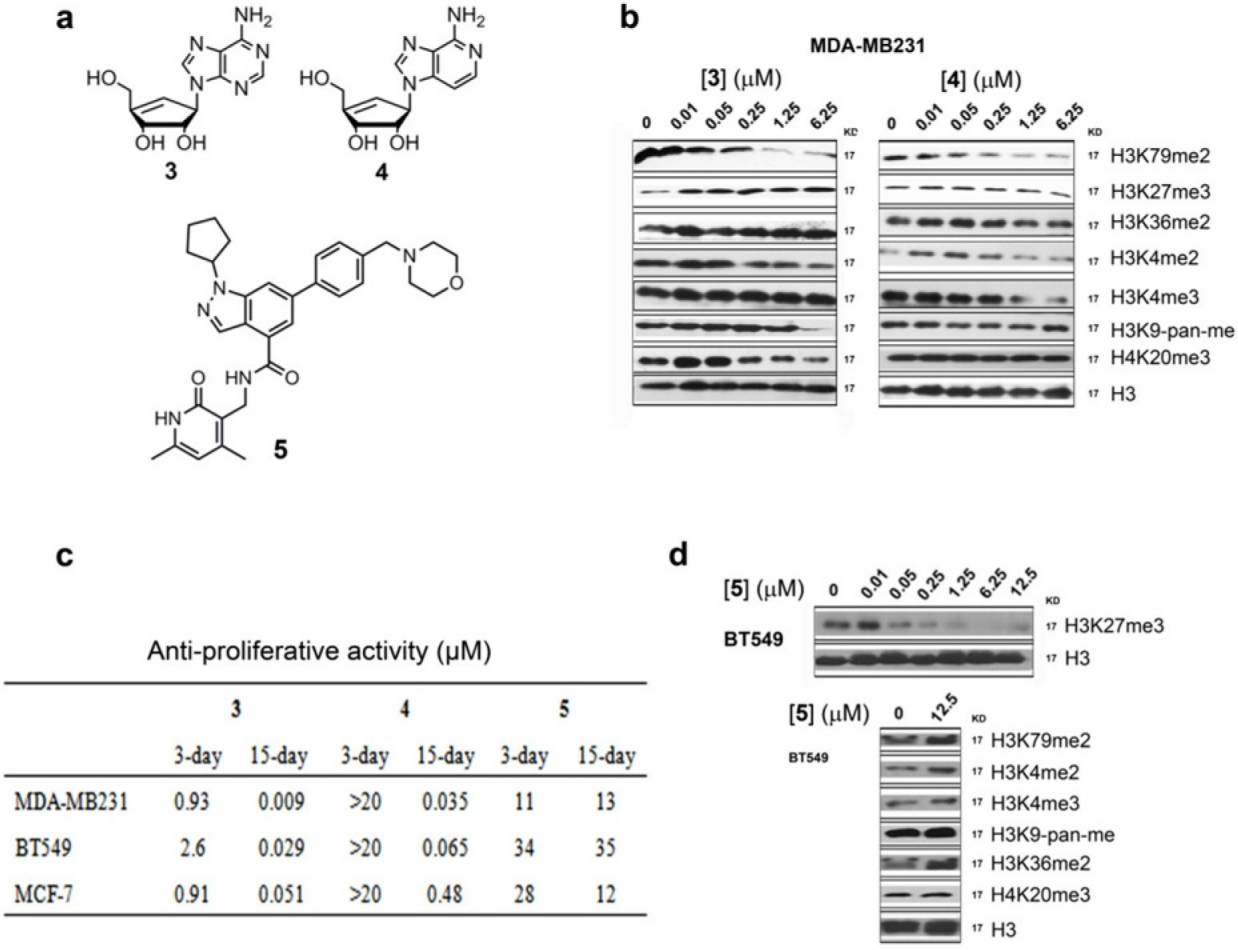
Activity of SAHH inhibitors **3** and **4** and EZH2 inhibitor **5** (**a**) Structures of compounds **3** – **5**; (**b**) Western blot results showed that compounds **3** and **4** selectively inhibited H3K79 methylation in MDA-MB231 with IC_50_ values of ~50 nM. These compounds had considerably weakened activity against methylation at other histone lysine residues; (**c**) Anti-proliferation activity of compounds **3** – **5** against breast cancer cell lines; (**d**) EZH2 inhibitor **5** only inhibited H3K27 methylation in BT549 cells.

We found that inhibition of S-adenosyl-homocycteine (SAH) hydrolase (SAHH) by neplanocin A (**3**) and its analog 3-deazaneplanocin A (**4**) (Fig. 4a) [32, 33] is an alternative approach to selective DOT1L inhibition. As illustrated in Supplementary Figure 7, DOT1L as well as all other histone/protein methyltransferases use S-adenosyl-methionine (SAM) as the enzyme cofactor and generate SAH upon completion of the methylation reaction. SAH is a strong, endogenous inhibitor of DOT1L with a *K*_i_ value of 160 nM [34, 35]. However, the inhibitory activity of SAH is considerably weaker against other lysine methyltransferases (*K*_i_ > 1 μM) [35]. To maintain cellular methylation, SAH is rapidly hydrolyzed by SAHH, which keeps a high cellular SAM:SAH ratio of ~40:1 [36]. Compound **3** (as well as **4**) potently inhibited SAHH with a *K*_i_ of 8.4 nM and caused a decreased SAM:SAH ration of up to 1:1.5 [32], which could effectively inhibit DOT1L (as well as other methyltransferases to a lesser extent). Compounds **3** and **4** were found to potently and selectively inhibit H3K79 methylation with IC_50_ values of ~50 nM (Fig. 4b). However, at concentrations of <1 μM, no or negligible inhibitory activities of **3** and **4** were observed against methylation at H3K4, H3K9, H3K27, H3K36 and H4K20 (Fig. 4b). Particularly, SAH was reported to be considerably less active against H3K27 methyltransferase EZH2 with a *K*_i_ of 7.5 μM [35]. Consistently with the biochemical data, the activities of compounds **3** and **4** against H3K27 methylation were found to be weak with IC_50_s of >6 μM (Fig. 4b).

### SAHH inhibitors exhibited potent activity against breast cancer cells

Compounds **3** and **4** were next tested for their activity against proliferation of the three DOT1L^+^ breast cancer cells. **3** exhibited moderate cytotoxicity in a 3-day treatment with EC_50_ values of 0.9–2.6 μM (Fig. 4c). This is because compound **3** can be phosphorylated at its 5′-OH in cells and inhibits cellular DNA/RNA synthesis [37]. Presumably due to the lack of the 3-N atom in the purine ring, compound **4** is less efficiently phosphorylated and showed no general cytotoxicity in a 3-day treatment with the EC_50_ values of >20 μM. For a 15-day treatment, compound **3** was found to possess very potent activity against MDA-MB231, BT549 and MCF-7 with EC_50_ values of 9, 29 and 51 nM, respectively, being 18 - 100× more active than the corresponding 3-day data. This is likely due to a synergistic (or additive) effect by the dual-role compound **3**, which possesses both cytotoxicity and selective H3K79 methylation inhibition. Compound **4** also exhibited potent anti-proliferation activity in a 15-day treatment with EC_50_ values of 35, 480 and 65 nM, showing again that H3K79 methylation inhibition can enormously enhance the killing efficacy of the compound. Moreover, the similarities between **1** and **4** in inhibiting H3K79 methylation and cell proliferation supported that the activity of compound **4** is mainly mediated by H3K79 methylation inhibition.

### EZH2/H3K27 methylation inhibition did not contribute to breast cancer killing

Compound **4** was previously reported to have activity against several cancer cells, including MCF-7 and MDA-MB231 [38, 39]. In these studies, the activity of **4** was mainly attributed to EZH2/H3K27 methylation inhibition. However, because of the large differences between the anti-proliferation activity (EC_50_: 35-480 nM) and H3K27 methylation inhibition (IC_50_ >6 μM) for **4**, our results supported that DOT1L/H3K79 methylation inhibition (with IC_50_ ~50 nM, Fig. 4b) is mainly responsible for the activity of compound **4** against these breast cancer cells.

To further probe the consequence of EZH2 inhibition in these breast cancer cells, a potent and specific EZH2 inhibitor, EPZ005687 (**5**, Fig. 4a) [40], was synthesized. With its reported *K*_i_ value of 24 nM against EZH2, compound **5** inhibited H3K27 methylation in the breast cancer cells with IC_50_s of ~50 nM (Fig. 4d). However, **5** exhibited only weak anti-proliferation activities against the three breast cancer cells with EC_50_ values of 11-35 μM (Fig. 4c) regardless of the treatment durations. These observations demonstrated that inhibition of EZH2/H3K27 methylation cannot significantly affect the proliferation of these breast cancer cells.

### Neplanocin A (3) potently inhibited CSC and metastatic potential

Compound **3** is of particular interest, because of the most potent anti-proliferation activity as well as the novel mode of action that combines H3K79 methylation inhibition with cytotoxicity in one compound. We believe a combination therapy should be more effective to treat DOT1L^+^ breast cancer, especially given the slow action of the DOT1L-specific inhibitors **1** and **2**. A potent cytotoxic drug may rapidly reduce the overall tumor burden to save more time for H3K79 methylation inhibition to correct cancer-relevant gene expression and inhibit CSCs, with the potential to eventually eliminate all cancer cells.

**Figure 5:**
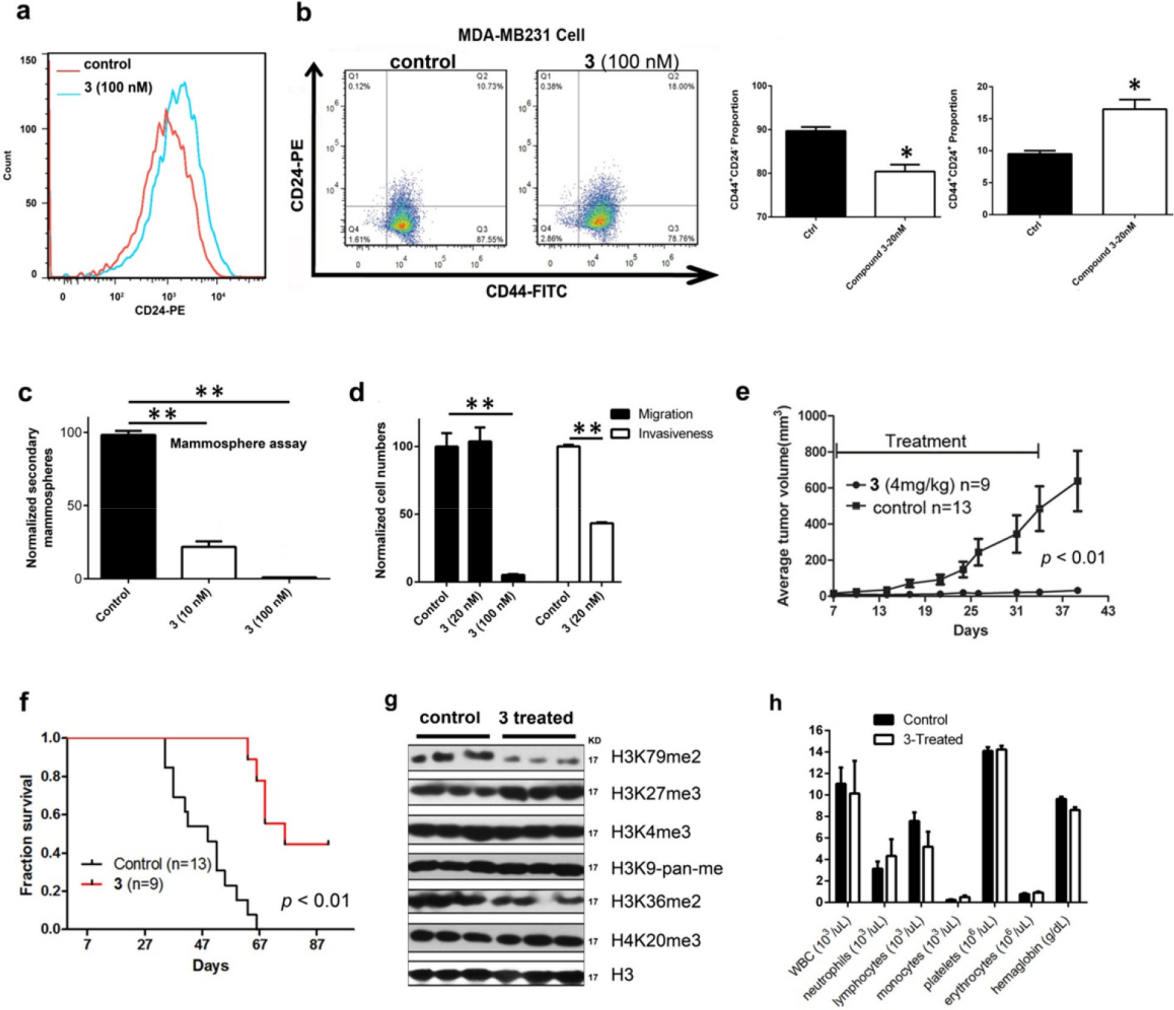
Biological activities of compound **3** against MDA-MB231 (**a**) Treatment with **3** caused increased cell population expressing high levels of CD24, showing this compound induced cell differentiation; (**b-d**) Treatment with **3** significantly (**b**) decreased the CD44^+^/CD24^−^ cell population and increased CD44^+^/CD24^+^ cell population, (**c**) inhibited secondary mammosphere formation, and (**d**) reduced cell migration and invasiveness. These results showed that **3** inhibited breast CSC-like cells and EMT; (**e**, **f**) Treatment with **3** (4 mg/kg/day for 28 days) significantly inhibited (**e**) tumor growth and (**f**) prolonged animal survivals in an MDA-MB231 xenograft mouse model; (**g**) In vivo treatment with **3** only significantly reduced H3K79 methylation levels in tumor tissues; (**h**) Treatment with **3** (4 mg/kg/day for 28 days) did not cause significant changes in blood cell counts and hemoglobin levels in mice (n = 3 for control and treatment groups). (Statistical analysis by Student's *t* test: *p < 0.05, **p < 0.01)

More biological activity testing of compound **3** was performed. Compound **3** (20 nM) was found to induce apoptosis of 12.1% MDA-MB231 cells on day 7, which was consistent with a cell cycle analysis showing an increased sub-G1 population (Supplementary Fig. 8a/b). Similar to DOT1L-specific inhibitors, **3** exhibited strong activities against breast CSCs. Drug treatment caused differentiation of MDA-MB231 CSC-like cells, resulting in a significantly expanded CD24^+^ cell population (Fig. 5a). Treatment with **3** also reduced the CD44^+^/CD24^−^ (Fig. 5b) and ALDH^+^ cell populations (Fig. 5c and Supplementary Fig. 8c). In addition, as compared to **1** and **2**, compound **3** showed more pronounced activity to inhibit formation of secondary mammospheres as well as cell migration and invasiveness of MDA-MB231 (Fig. 5d). The increased potency of compound **3** in these assays could be attributed to the synergistic (or additive) effect for the dual-role compound.

### Neplanocin A (3) exhibited potent in vivo antitumor activity without overt toxicity

Because DOT1L inhibitors **1** and **2** have poor pharmacokinetics with short plasma half-lives [16], we did not examine the in vivo antitumor activity of these compounds. Rather, due to its extremely potent activity as well as novel mechanism of action, compound **3** was evaluated for the in vivo antitumor activity using an MDA-MB231 xenograft mouse model. 10^6^ cells per mouse were injected into the mammary fat pads of athymic nude mice and palpable tumors (with diameters of ~3 mm) were observed in ~7 days. Compound **3** was administered in a dose of 4 mg/kg/day for 28 days using an osmotic pump implanted subcutaneously in each mouse. This administration route was chosen because previous studies showed the importance of constant H3K79 methylation inhibition [41]. As compared to the tumors (n = 13) in the control group, the treatment (n = 9) caused tumor growth inhibition of >95% (Fig. 5e), together with significantly prolonged animal survivals (Fig. 5f). Four tumors (out of nine) in the treatment group were found to exhibit tumor regression during the treatment and remained to be small (1-3 mm in diameter) in the end of the experiment. In a separate experiment, three mice each from the control and treatment groups were sacrificed on day 14 and tumor tissues were extracted. Western blot experiments for the freshly separated cells showed only H3K79 methylation was significantly inhibited by the treatment with compound **3** (Fig. 5g). These results were consistent with the cell based experiments. In addition, the compound was found to cause no obvious toxicities: no significant changes in blood cell counts, hemoglobin (Fig. 5h) and body weight were observed on day 28. These results showed that compound **3** could be a promising drug candidate for the treatment of breast cancer.

## DISCUSSION

Breast cancer has posed a major threat to women's health, accounting for ~25% of all female cancers and causing ~440,000 deaths per year worldwide. Breast cancer patients rarely die of the local tumors, with >90% of the mortalities being due to metastasis and drug resistance followed by tumor recurrence. CSCs are believed to be responsible for breast cancer metastasis and recurrence, because of their drug resistance and association with EMT. Discovery of compounds that have selective activity against CSCs is therefore of importance.

Critical roles of histone methylation in cancer have projected the perspective that selective modulators of histone methylation could be novel probes for cancer biology studies as well as clinically useful therapeutics. Although development of such compounds has still been in its infancy [4], activities of DOT1L inhibitors (e.g., compounds **1** and **2**) against MLL-rearranged leukemia (16–18, 41) and EZH2 inhibitors (e.g., compound **5**) against EZH2-mutated lymphoma [40] have demonstrated the potential as well as ample opportunities for this direction.

In the present study, bioinformatics analysis of a large breast cancer genetic database has implicated that DOT1L plays an important role in the malignancy. DOT1L-specific inhibitors **1** and **2** were found to have strong activity against proliferation of DOT1L^+^ breast cancer cells with EC_50_ values as low as 190 nM, but these compounds were inactive against DOT1L^−/low^ breast or non-breast cancer cells. Comparative studies of the microarray data between siRNA and compounds **1**/**2** indicated the activity of these compounds is mediated by DOT1L inhibition, but not due to off-target effects. These results showed that DOT1L is a novel drug target for DOT1L^+^ breast cancer. Given ~50% breast cancers in the clinic express relatively high levels of DOT1L (Supplemental Fig. 2), this finding could have a high impact to breast cancer research and treatment.

Mechanistically, anti-proliferation activity of the DOT1L inhibitors is not due to cytotoxicity. By inhibiting H3K79 methylation, a key “histone code” for active gene transcription, compounds **1** and **2** were able to down-regulate expression of the genes that are important to breast cancer growth (e.g., pro-proliferation genes in PAM50, Fig. 3d/e). In addition, DOT1L inhibition can down-regulate expression of EMT genes and reduce in vitro cell migration and Matrigel invasion, showing the potential to inhibit tumor metastasis. The characteristic, slow action of the DOT1L inhibitors could be due to a long time needed for a series of cellular events that lead to proliferation arrest, including H3K79 methylation inhibition (with the maximal effect occurring in ~4 days) followed by decreased mRNA expression of the target genes, as well as ultimately the depletion of the gene products (proteins) key to cancer proliferation. Moreover, despite the importance of DOT1L in maintaining hematopoiesis [42, 43], pharmacological inhibition of DOT1L did not cause overt toxicities (shown in this and previous studies [16, 41]), indicating that there could be a sufficient therapeutic window for clinical applications.

In addition, inhibition of SAHH was found to be an alternative approach to selective inhibition of H3K79 methylation, because SAH is a more potent, endogenous inhibitor of DOT1L than other lysine methyltransferases [34, 35]. Indeed, our experiments showed that potent SAHH inhibitors **3** and **4** selectively decreased H3K79 methylation with IC_50_ of ~50 nM. However, these compounds had negligible effects on other lysine methylation sites at concentrations of <1 μM. Similar to DOT1L-specific inhibitors **1** and **2**, compound **4** also exhibited strong anti-proliferation activity against DOT1L^+^ breast cancer cells, mainly because of H3K79 methylation inhibition. Compound **3**, a dual-role compound that is both a selective H3K79 methylation inhibitor and a cytotoxic agent, was found to have the most potent antitumor activity in vitro and in vivo, showing that the combination therapy could be a viable approach to breast cancer treatment.

The activity of compound **4** was previously attributed to EZH2 inhibition, likely due to the importance of EZH2-containing polycomb repressive complex 2 (PRC2) as well as the observation of EZH2-overexpression in many types of cancers [44–46]. However, the weak activity of SAH against EZH2 as well as the weak inhibition of H3K27 methylation by **4** indicated that this is less likely to be the case. This point was confirmed by the weak anti-proliferation activity of the potent EZH2 inhibitor **5**, showing that inhibition of H3K27 methylation alone was not sufficient to inhibit proliferation of these cancer cells. Presumably, other proteins such as DNA methyltransferase [47, 48] also participate in PRC2-mediated gene repression. Therefore, inhibition of EZH2/H3K27 methylation may not be sufficient to correct the overall effects by PRC2-mediated gene silencing in these cancer cells.

## METHODS

### Chemical probes

Compounds **1** – **5** were synthesized using the published methods [49, 17, 50, 51, 40] and characterized by ^1^H and ^13^C NMR on a Varian (Palo Alto, CA) 400-MR spectrometer, showing identical NMR spectra to those reported. The compound purities were determined by a Shimadzu Prominence HPLC with a Zorbax C18 column (4.6 × 250 mm) monitored by UV absorbance at 254 nm. The purities of all compounds were found to be >95%.

### Cell culture, antibodies and RNAi

Human cell lines BT549, MDA-MB231, MCF-7, MDA-MB157, HCC70, A549 and NB4 were obtained from the American Type Culture Collection (ATCC) and cultured using ATCC recommended conditions. Primary antibodies against H3K79me2, H3K27me3, H3K4me3, H3K36me2, H4K20me3, H3K9me-pan were purchased from Cell Signaling (Danvers, MA). siRNA experiments were performed by transfecting MDA-MB-231 cells with X-tremeGENE Transfection Reagent (Roche) and 10 nM siRNA (Invitrogen), according to the manufacturer's protocol. The sequences for DOT1L siRNA (Invitrogen) were:

siRNA-1: 634-CGCGAGUUCAGGAAGUGGAUGAAAU;
siRNA-2: 117-CGAUAAACAUCACGAUGCUGCUCAU;
siRNA-3: 104-CGCUGCCGGUCUACGAUAAACAUCA;
siRNA-4: 470-CCCAGAUGAUUGAUGAGAUCAAGAU;
siRNA-5: 120-UAAACAUCACGAUGCUGCUCAUGAA.
siRNA-`mock represents an siRNA that has no homology to any vertebrate transcriptome.

### Western blot

10^6^ cells/well were incubated with increasing concentrations of a compound for 5 days and histones extracted with the EpiQuik™ Total Histone Extraction Kit (Epigentek) according to the manufacturer's protocol. Equal amounts of histones (2μg) were separated on SDS-PAGE and transferred to PVDF membranes. The blots were probed with primary antibodies, followed by anti-rabbit IgG (Thermo Scientific) secondary antibodies. For western blot analysis of MDA-MB231 xenograft tumors, tumors were harvested and frozen in liquid nitrogen. Histones were extracted and analyzed by immunoblotting as described above.

### Analysis of cell proliferation, apoptosis and cell cycle

2,500 or 5,000 cells/well in 96-well plates were incubated with increasing concentrations of a compound for 3 or 15 days, with media and compounds refreshing every 3-4 days. Cell viability was evaluated using an MTT assay. The EC_50_ values in Table 1 were the mean values from at least three independent experiments.

For apoptosis and cell cycle analysis, 10^5^ cells/well were incubated with increasing concentrations of a compound for 7 or 10 days. Apoptosis was determined using the FITC Annexin V Apoptosis Detection Kit I (BD Bioscience) according to the manufacturer's protocol. Cells for cell cycle analysis were fixed in cold 70% ethanol at 4°C and stained with Propidium iodide (Sigma-Aldrich) (20 μg/ml) and RNase A (10 μg/ml) for 30min. Cells were analyzed using a FACS Calibur (BD Biosciences/Applied Biosystems) and data were processed using the program Flowjo (version7.6.5).

### Quantitative real-time PCR

10^4^ cells/well were incubated with compounds for 14 days with media containing compounds replaced every 3-4 days. Total RNA was extracted from cells using RNeasy mini kit (Qiagen). 100-1000 ng of total RNA was reverse transcribed using iScript™ Reverse Transcription Supermix (Bio-Rad) according to the manufacturer's protocol. Quantitative real-time PCR was carried out using the RT^2^ SYBR Green ROX™ qPCR Mastermix (Qiagen) according to the manufacturer's instructions. Measurements were performed in triplicate, using GAPDH or human 18S ribosomal RNA as a reference gene. The sequences of primers used are listed in the Supplementary Table. Real-time PCR was performed using the ABI Prism 7300 sequence detection system.

### In vitro migration and invasion assay

5 × 10^4^ cells/well were incubated with compounds for 14 days. For transwell migration assays, 5× 10^4^ cells were plated in the top chamber with the non-coated membrane (24-well insert; pore size, 8 mm; BD Biosciences). For invasion assays, 5× 10^4^ cells were plated in the top chamber with Matrigel-coated membrane. The cells on the apical side of each insert were then scraped off after 22 h for both assays. The wells were washed with PBS, fixed with 100% methanol and stained with DAPI. Cells that had migrated to the basal side of the membrane were visualized and counted with a Nikon Eclipse TE2000-U microscope at 10×. Pictures of three random fields were obtained and the number of cells that had migrated quantified using ImageJ analysis software.

### ALDEFLUOR assay and flow cytometry

5 × 10^4^ cells/well were incubated with compounds for 14 days. The ALDH activity of cells was determined by using the ALDEFLUOR assay kit (StemCell Technologies) according to the manufacturer's instructions. Combinations of fluorochrome-conjugated monoclonal antibodies against human CD44-FITC and CD24-PE were obtained from BD Biosciences. Primary antibodies or the respective isotype controls (BD Biosciences) were added to the cell suspension, according to the manufacturer's recommendation, and incubated at 4°C for 20 min. Dead cells were eliminated using propidium iodide (Sigma). The labelled cells were analyzed on a FACS Calibur (BD Biosciences/Applied Biosystems).

### Mammosphere assay

The protocol for mammosphere assay was performed using a published method [28]. The numbers of secondary mammospheres in each well were counted using a Leica MZ12.5 microscope.

### MDA-MB231 xenograft mouse model and drug treatment

Female athymic nude mice (4 to 6 weeks old) were obtained from Charles River (Wilmington, MA, USA) and maintained under pathogen-free conditions. The use of athymic nude mice and their treatment were approved by the BCM Institutional Animal Care and Use Committee, and the experiments were conducted in strict compliance with the regulations. 2 ×10^6^ MDA-MB-231 cells were injected into the upper right mammary fat pad of the recipient mouse and tumor growth and animal weights were monitored twice a week. The tumor volumes were calculated using the formula length × (width)^2^/2. When the tumors reached a size of about 20–30 mm^3^, mice were randomly segregated into control and treatment groups. Mice in the treatment group were implanted subcutaneously an osmotic pumps (Alzet model 2004) containing 2.8 mg of **3** in 200 μL of PBS. Animals were sacrificed when the volumes of tumors reaching ~1.5 cm^3^. Log-rank analysis was used to determine statistical significance of the survival curves using Prism 5.0 (GraphPad).

### RNA Amplification and Microarray Data Analysis

10^5^ cells/well were incubated with compounds for 14 days. Cells were lysed immediately in TRIzol® Reagent, frozen in liquid nitrogen, and shipped to Asuragen, Inc. (Austin, TX) for microarray experiments. RNA was isolated, amplified, and hybridized to Illumina Human HT-12 v4 arrays according to the manufacturer's protocol. Microarray data were log2 transformed and then normalized to have the same median for all the arrays. Moderate *t*-statistics were used to test if genes were differentially expressed between the groups of interest. Benjamini and Hochberg method was used to correct for multiple comparisons. R and Bioconductor packages were used for all the statistical analyses (http://cran.us.r-project.org/, http://www.bioconductor.org/). Gene set enrichment analysis was performed using GSEA software from Broad Institute (Boston, MA).

### Statistical analysis

Student's *t*-test was applied to calculate the statistical significance (*p* value) for all data (except for microarray analysis) using Prism 5.0 (GraphPad). Statistical analysis for microarray studies is described in the Microarray section above.

## SUPPLEMENTARY FIGURES AND TABLES



## ABBREVIATIONS

ALDH: aldehyde dehydrogenase
CSC: cancer stem cells
DOT1L: disruptor of telomeric silencing 1 like
EMT: epithelial mesenchymal transition
FACS: fluorescence-activated cell sorting
GSEA: Gene set enrichment analysis
H3K27: histone H3-lysine27
H3K79: histone H3-lysine79
MLL: mixed lineage leukemia
PRC2: polycomb repressive complex 2
SAHH: S-adenosylhomocysteine hydrolase
siRNA: small interfering RNA
