# Supplementary material for: Inhibition of histone H3K79 methylation selectively inhibits proliferation, self-renewal and metastatic potential of breast cancer
Source: Oncotarget. 2014 Nov 10;5(21):10665–77. doi: 10.18632/oncotarget.2496 (PMC4279401; doi:10.18632/oncotarget.2496)
Supplement: Supplementary file 1 [file oncotarget-05-10665-s001.pdf]

SUPPLEMENTARY FIGURES AND TABLE

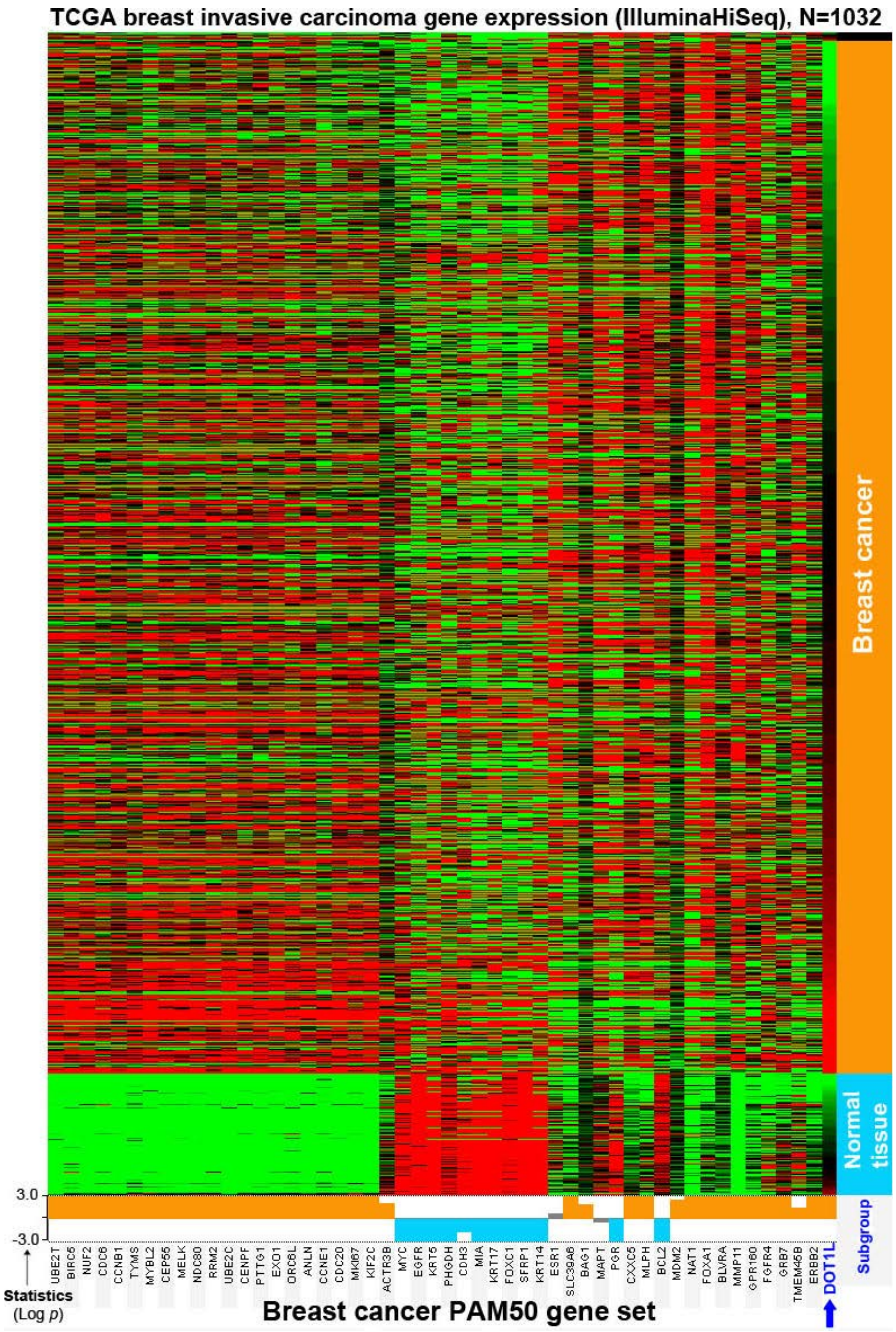

Supplementary Figure S1: Heat map of the expression levels of DOT1L (marked with a blue arrow) and PAM50 gene set in breast cancers (N=835) and normal breast tissues (N=100), showing that overexpression of DOT1L correlates with breast cancer (the orange subgroup) with a p value of <0.001. (The data and figure were generated by UCSC cancer browser, Zhu, et al. 2009).

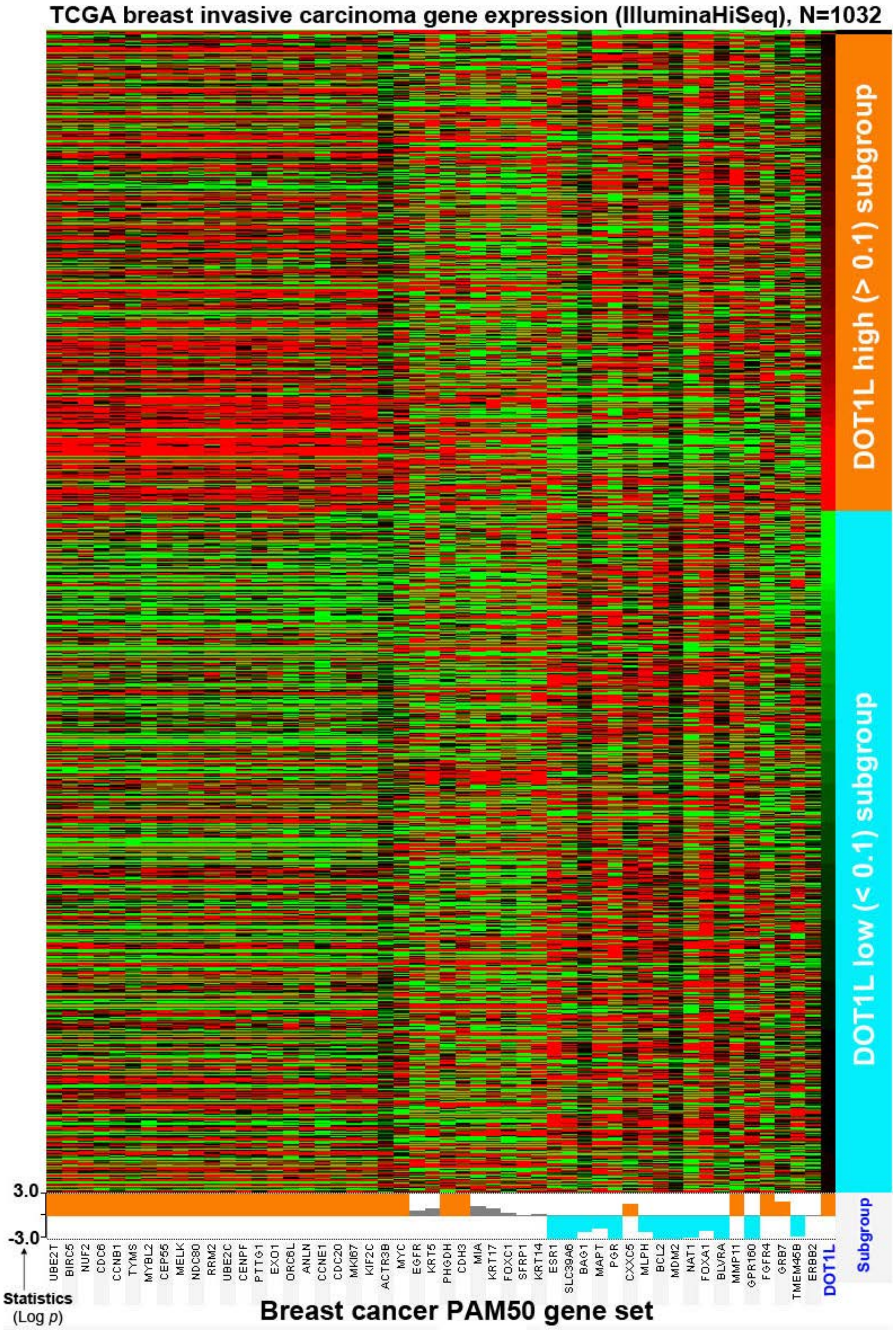

Supplementary Figure S2: Heat map of the expression levels of DOT1L and PAM50 gene set in the same database as that of Supplementary Fig. 1, showing that overexpression of DOT1L correlates with that of the pro-proliferation genes in the left-panel of the PAM50 gene set (p <0.001), whose overexpression links to high proliferation and poor prognosis for breast cancer patients.

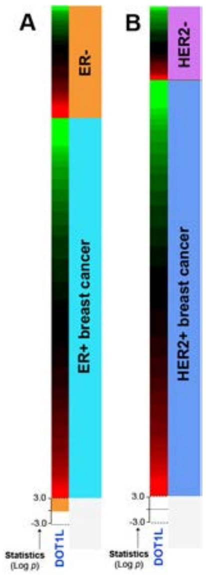

Supplementary Figure S3: Heat map of the expression levels of DOT1L in the same database as that of Supplementary Fig. 1, showing that **(A)** higher levels of DOT1L are correlated with ER^- breast cancer (p <0.001) and **(B)** higher levels of DOT1L are not correlated with HER2 status.

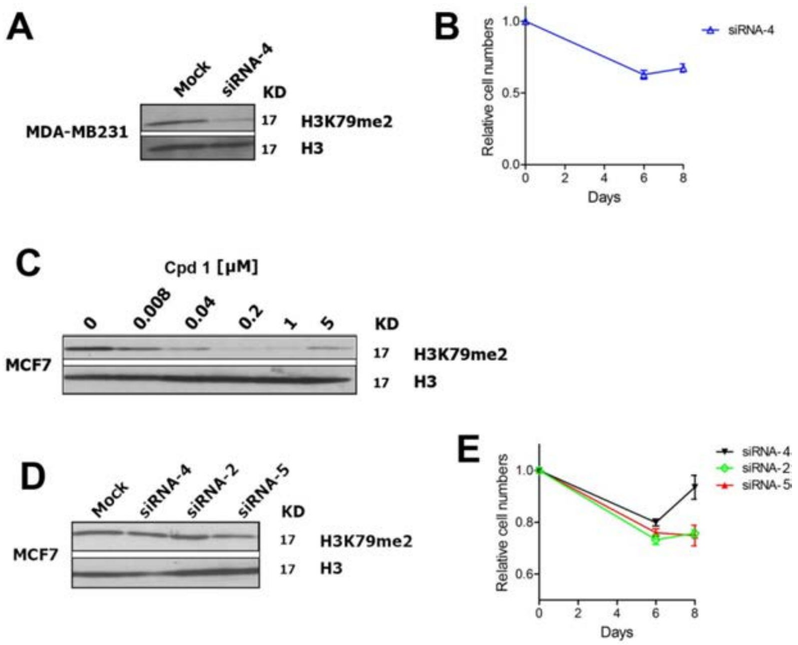

Supplementary Figure S4: Effects of DOT1L inhibition in MDA-MB231 (ER$^-$) and MCF-7 (ER$^+$) breast cancer cells. (A) Treatment of MDA-MB231 cells with siRNA-4 significantly reduced H3K79 methylation and **(B)** inhibited cell proliferation; **(C)** Treatment of MCF-7 cells with compound **1** significantly reduced H3K79 methylation in a dose-dependent manner; **(D)** Treatment of MCF-7 cells with three siRNAs also reduced H3K79 methylation and **(E)** inhibited cell proliferation. (Statistical analysis by Student's *t* test: $p < 0.01$ for all experiments, except for MCF-7 cells with siRNA-2 on day 8)

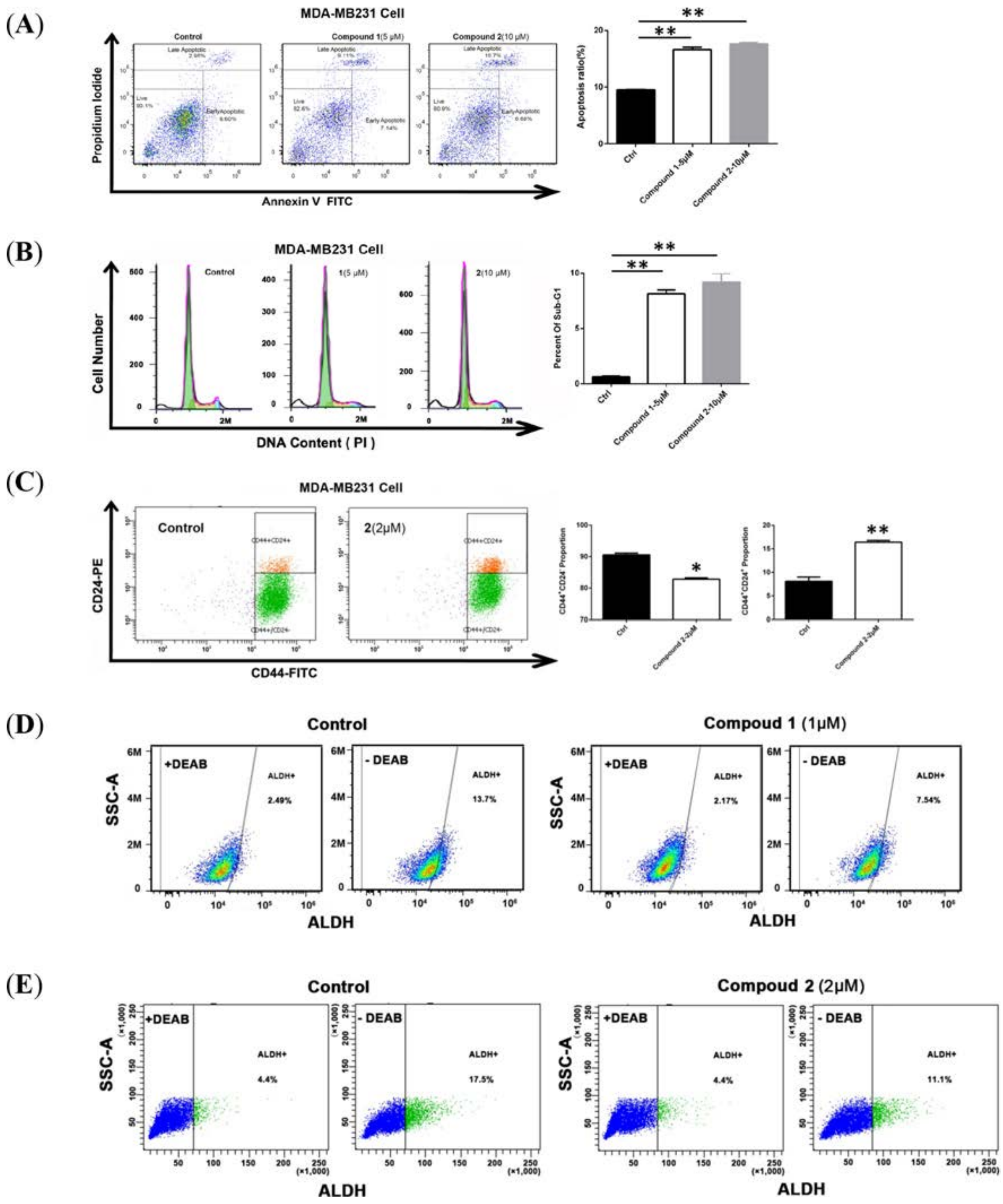

Supplementary Figure S5: Effects of DOT1L inhibition in MDA-MB231 cells. **(A)** Treatment with compound **2** induced apoptosis in 7.8% MDA-MB231 cells; **(B)** Treatment with 2 caused an increased sub-G1 phase cell population; **(C)** Treatment of MDA-MB231 with compound **2** significantly decreased the $CD44^{+}/CD24^{-}$ cell population; **(D and E)** Treatment of MDA-MB231 with compounds ^{1} and ^{2} reduced the $ALDH^{+}$ cell population. (Statistical analysis by Student's *t* test: *$p < 0.05$, **$p < 0.01$)

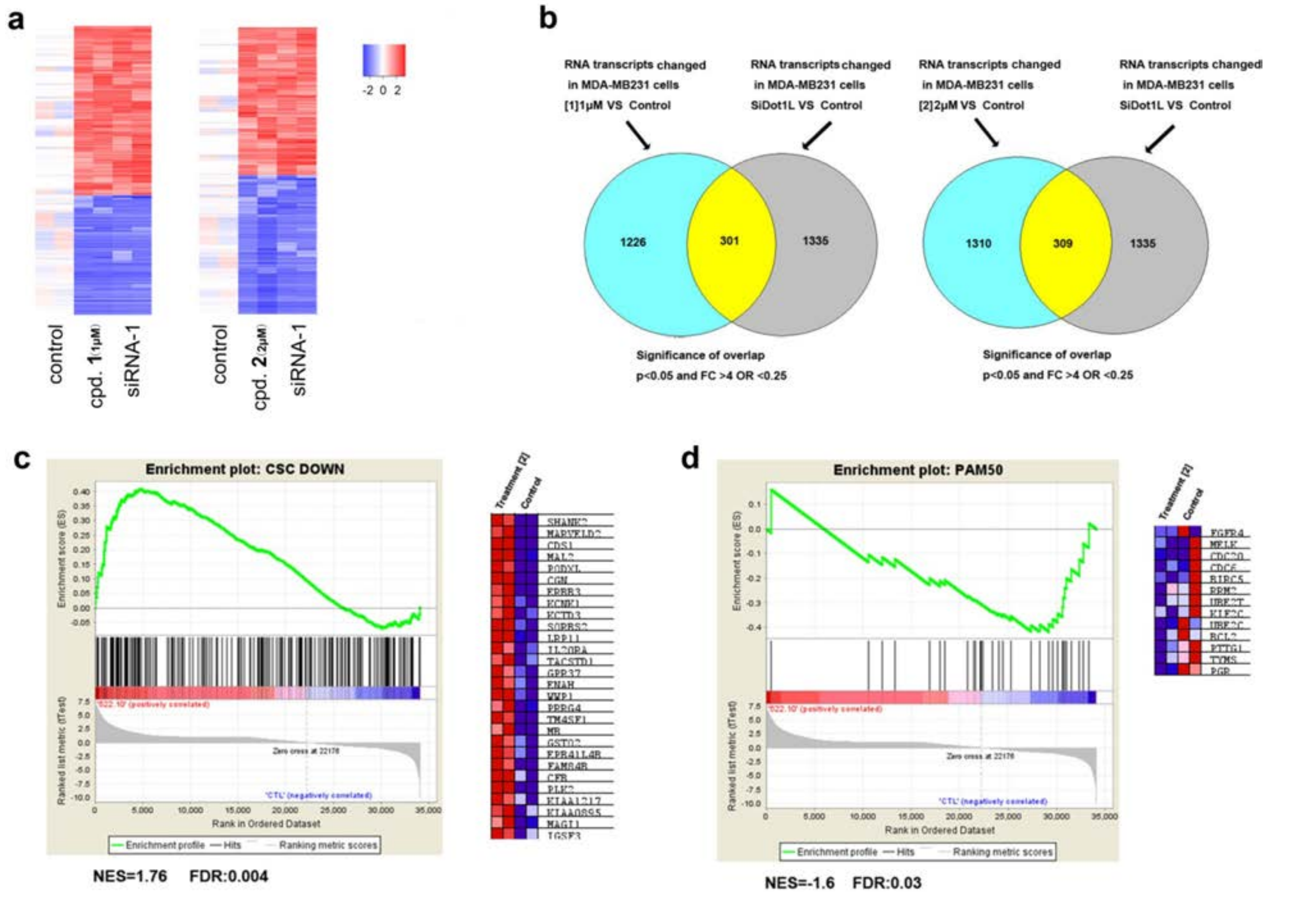

Supplementary Figure S6: Microarray results of DOT1L inhibition in MDA-MB231 cells. (a) Heat maps of the overlapping upregulated and downregulated genes between samples treated with compounds **1** (1 µM) or **2** (2 µM) and those with siRNA-1 (duplicate samples, $p < 0.05$, fold change > 4); **(b)** Venn diagram showing numbers of significantly changed genes between samples treated with **1** or **2** and those with siRNA-1 (p = 1E-14 or 1E-16, respectively, one-sided Fisher's exact test); **(c, d)** Upon treatment with compound **2** (10 µM), GSEA plots showed **(c)** upregulation of the genes whose expression is low in breast CSCs and **(d)** downregulation of the pro-proliferation genes in the PAM50 gene set. The right panels in **(c)** and **(d)** are heat maps showing expression for selected genes in the leading edges of the GSEA plots.

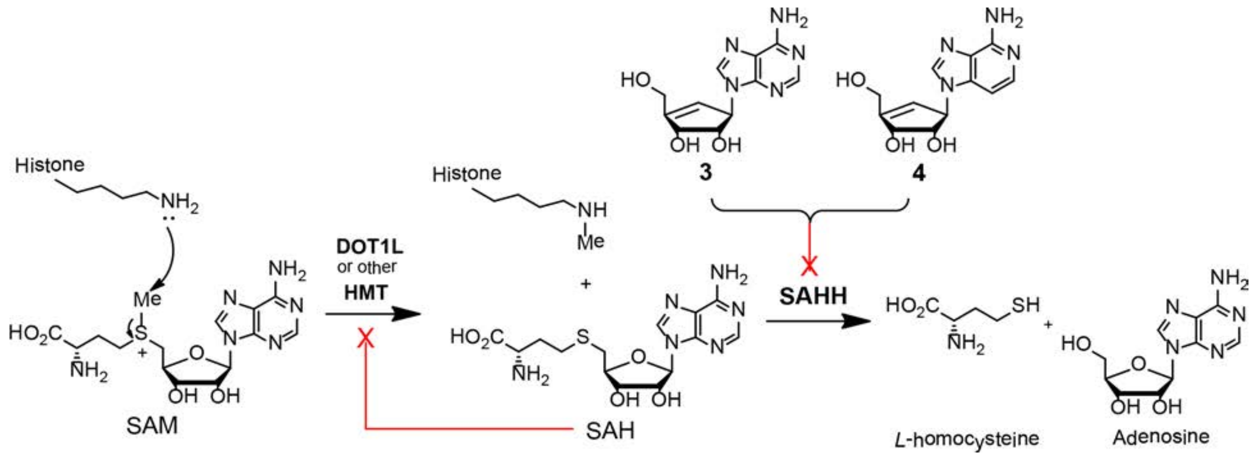

Supplementary Figure S7: Mechanisms of DOT1L (as well as other HMTs) and SAHH, showing how SAHH inhibitors indirectly inhibit DOT1L. SAH, the product of DOT1L catalyzed reaction, is a strong inhibitor (K_i = 160 nM) of DOT1L. Inhibition of SAHH by compounds **3** and **4** caused an increased cellular concentration of SAH, resulting in strong DOT1L/H3K79 methylation inhibition. Due to considerably decreased inhibitory activity of SAH against other HMTs (K_i > 1 µM; e.g., K_i of 7.5 µM against EZH2), **3** and **4** had less activity against other HMTs as well as methylation at other histone lysine residues.

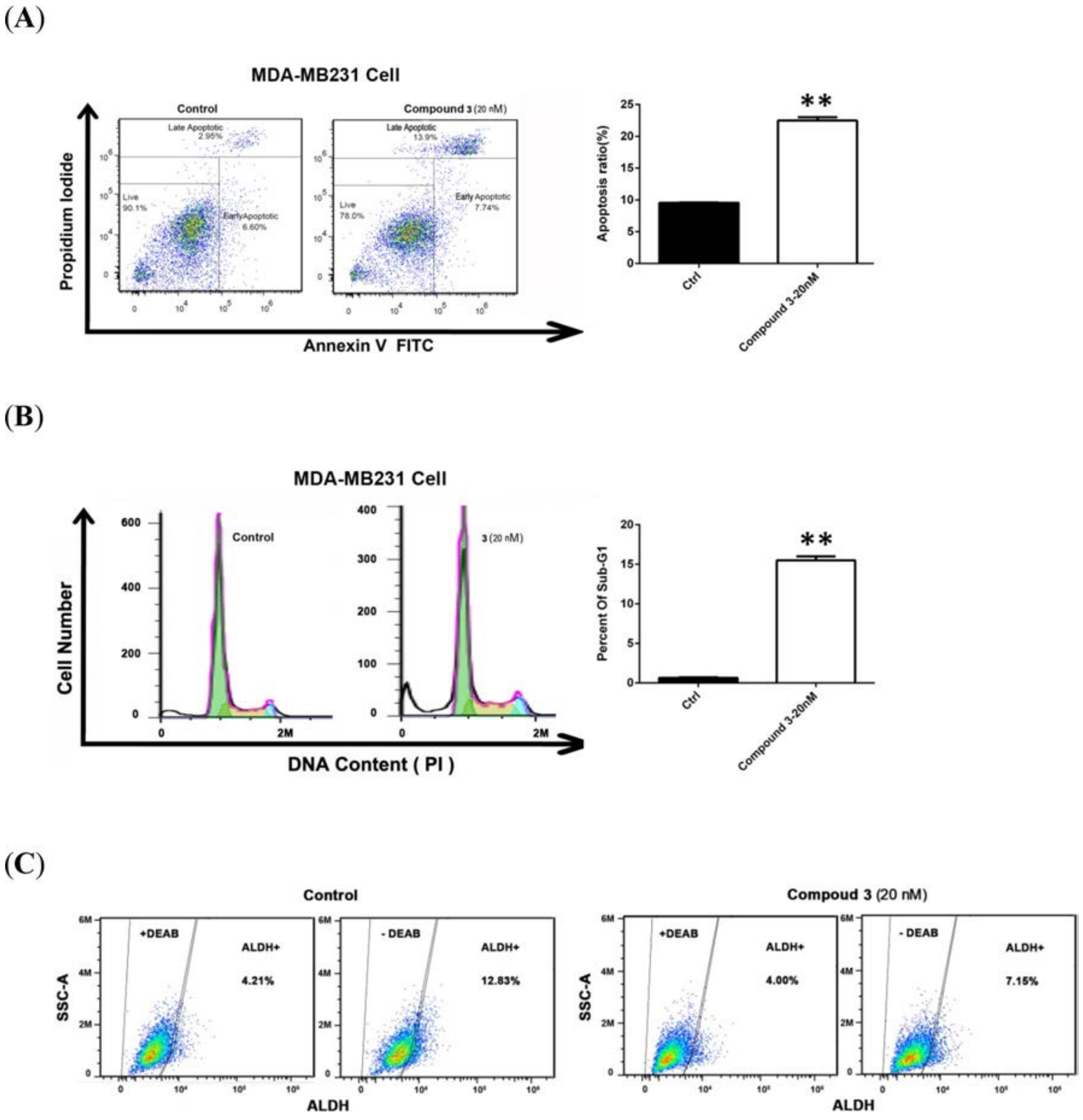

Supplementary Figure S8: Activities of compound 3 on MDA-MB231 cells. **(A)** Treatment with compound **3** induced apoptosis in 12.1% MDA-MB231 cells; **(B)** Treatment with **3** caused an increased sub-G phase cell population (from 4.4% to 21.2%). (Statistical analysis by Student's *t* test: $*p < 0.05$, $**p < 0.01$); **(C)** Treatment of MDA-MB231 with compound **3** for 15 days decreased the $ALDH^+$ cell population.

Supplementary Table S1. Primer sequences for Q-PCR

Gene Name	Forward Primer	Revserse Primer
E-Cadherin	CCCAATACATCTCCCTTCACAG	CCACCTCTAAGGCCATCTTTG
GAPDH	ATCAAGTGGGGCGATGCTG	ACCCATGACGAACATGGGG
ZEB-1	ACCCTTGAAAGTGATCCAGC	CATTCCATTTTCTGTCTTCCGC
SNAI1	GGAAGCCTAACTACAGCGAG	CAGAGTCCCAGATGAGCATTG
TGFB2	CTGATCCTGCATCTGGTCACG	TGGGGGACTGGTGAGCTTC
CCNB1	AATAAGGCGAAGATCAACATGGC	TTTGTTACCAATGTCCCCAAGAG
MYBL2	TCTGGCTCTTGACATTGTGG	TCAGGACAAGATGAGGGTCC
CENPF	CTCTCCCGTCAACAGCGTTC	GTTGTGCATATTCTTGGCTTGC
CCNE1	GCCAGCCTTGGGACAATAATG	CTTGCACGTTGAGTTTGGGT
CDC20	GACCACTCCTAGCAAACCTGG	GGGCGTCTGGCTGTTTTCA
UBE2C	TGATGTCTGGCGATAAAGGGATT	GTGATAGCAGGGCGTGAGGAA
PTTG1	ACCCCTCAAACAAAAACAGCC	GGCAGGAACAGAGCTTTTTGC
18S rRNA	GAATGAGTCCACTTTAAATCCT	CAAGATCCAACTACGAGCTTTT
